# Machine learning algorithms to the early diagnosis of fetal alcohol spectrum disorders

**DOI:** 10.3389/fnins.2024.1400933

**Published:** 2024-05-06

**Authors:** Anna Ramos-Triguero, Elisabet Navarro-Tapia, Melina Vieiros, Afrooz Mirahi, Marta Astals Vizcaino, Lucas Almela, Leopoldo Martínez, Óscar García-Algar, Vicente Andreu-Fernández

**Affiliations:** ^1^Grup de Recerca Infancia i Entorn (GRIE), Institut d'investigacions Biomèdiques August Pi i Sunyer (IDIBAPS), Barcelona, Spain; ^2^Department de Cirurgia i Especialitats Mèdico-Quirúrgiques, Universitat de Barcelona, Barcelona, Spain; ^3^Instituto de Investigación Hospital Universitario La Paz (IdiPAZ), Hospital Universitario La Paz, Madrid, Spain; ^4^Faculty of Health Sciences, Valencian International University (VIU), Valencia, Spain; ^5^Department of Neonatology, Instituto Clínic de Ginecología, Obstetricia y Neonatología (ICGON), Hospital Clínic-Maternitat, BCNatal, Barcelona, Spain; ^6^Department of Pediatric Surgery, Hospital Universitario La Paz, Madrid, Spain; ^7^Biosanitary Research Institute, Valencian International University (VIU), Valencia, Spain

**Keywords:** fetal alcohol spectrum disorders, machine learning, eXtreme Gradient Boosting (XGB), Random Forest (RF), neurodevelopment, PAE, early diagnosis

## Abstract

**Introduction:**

Fetal alcohol spectrum disorders include a variety of physical and neurocognitive disorders caused by prenatal alcohol exposure. Although their overall prevalence is around 0.77%, FASD remains underdiagnosed and little known, partly due to the complexity of their diagnosis, which shares some symptoms with other pathologies such as autism spectrum, depression or hyperactivity disorders.

**Methods:**

This study included 73 control and 158 patients diagnosed with FASD. Variables selected were based on IOM classification from 2016, including sociodemographic, clinical, and psychological characteristics. Statistical analysis included Kruskal-Wallis test for quantitative factors, Chi-square test for qualitative variables, and Machine Learning (ML) algorithms for predictions.

**Results:**

This study explores the application ML in diagnosing FASD and its subtypes: Fetal Alcohol Syndrome (FAS), partial FAS (pFAS), and Alcohol-Related Neurodevelopmental Disorder (ARND). ML constructed a profile for FASD based on socio-demographic, clinical, and psychological data from children with FASD compared to a control group. Random Forest (RF) model was the most efficient for predicting FASD, achieving the highest metrics in accuracy (0.92), precision (0.96), sensitivity (0.92), F1 Score (0.94), specificity (0.92), and AUC (0.92). For FAS, XGBoost model obtained the highest accuracy (0.94), precision (0.91), sensitivity (0.91), F1 Score (0.91), specificity (0.96), and AUC (0.93). In the case of pFAS, RF model showed its effectiveness, with high levels of accuracy (0.90), precision (0.86), sensitivity (0.96), F1 Score (0.91), specificity (0.83), and AUC (0.90). For ARND, RF model obtained the best levels of accuracy (0.87), precision (0.76), sensitivity (0.93), F1 Score (0.84), specificity (0.83), and AUC (0.88). Our study identified key variables for efficient FASD screening, including traditional clinical characteristics like maternal alcohol consumption, lip-philtrum, microcephaly, height and weight impairment, as well as neuropsychological variables such as the Working Memory Index (WMI), aggressive behavior, IQ, somatic complaints, and depressive problems.

**Discussion:**

Our findings emphasize the importance of ML analyses for early diagnoses of FASD, allowing a better understanding of FASD subtypes to potentially improve clinical practice and avoid misdiagnosis.

## 1 Introduction

Fetal alcohol spectrum disorder (FASD) is a range of neurodevelopmental impairments produced by prenatal alcohol exposure (PAE) (Hoyme et al., 2005; Popova et al., 2023). Epidemiological studies estimate a global prevalence of 0.77% (Lange et al., 2017), with regional variations observed, particularly in Europe and North America where prevalence ranges from 2.0 to 5.0% (Wozniak et al., 2019). Despite its prevalence, FASD remains underdiagnosed due to the wide variety of associated symptoms and the complexity in the diagnosis of some of them specifically to FASD, which can overlap with alternative diagnoses, such as attention deficit hyperactivity disorder (ADHD). In addition, the social stigma can pose significant challenges for affected individuals, their families and healthcare systems.

Individuals diagnosed with FASD face a wide range of neurocognitive impairments and social challenges that persist throughout their lives (Kelly et al., 2000; Champagne et al., 2023). Primary disabilities associated with FASD include impairments in adaptive functioning, memory, attention, abstract thinking, judgement, and cause-effect reasoning (Maya-Enero et al., 2021). Secondary disabilities, which result from the interaction of primary disabilities with environmental factors, can adversely affect an individual's ability to actively and positively participate in their lives and can lead to academic failure, low self-esteem, housing instability, and depression (Pei et al., 2011; Leenaars et al., 2012). The complex interplay between these cognitive impairments and social difficulties highlights the need for early comprehensive diagnostic strategies to adequately support affected individuals.

The diagnostic criteria for FASD are multifaceted and include four domains: PAE, facial features, growth, and neurodevelopment (Hoyme et al., 2016). These domains create a spectrum from the most severe condition of FASD, the fetal alcohol syndrome (FAS) to alcohol-related brain damage (ARBD), with partial FAS (pFAS) and alcohol-related neurodevelopmental disorder (ARND) as intermediate terms. Several guidelines are commonly used, including those from the Institute of Medicine, Canadian Guidelines, Centers for Disease Control (CDC), and the University of Washington's 4-digit code (Bastons-Compta et al., 2016; Maya-Enero et al., 2021). The Institute of Medicine (IOM) criteria, which include craniofacial anomalies, growth retardation, mental disabilities and developmental disorders, are currently recommended for diagnosis (Hoyme et al., 2016). However, this approach has limitations, such as difficult physical assessments, extensive neuropsychological assessments and underreporting of alcohol use during pregnancy. Obtaining a confirmed history of alcohol use during pregnancy is hampered by various factors, such as change of custody or maternal death. Consequently, a significant proportion of individuals with FASD remain undiagnosed or receive delayed diagnoses, exacerbating their difficulties and limiting their access to early interventions and support services (Jańczewska et al., 2019).

The search for novel strategies and methodologies for early diagnosis is one of the most promising fields of research in FASD. Timely identification of affected individuals is crucial for implementing personalized interventions and mitigating the long-term impact of the disorder on cognitive, social, and behavioral outcomes. In this context, emerging technologies, such as machine learning, offer promising avenues for improving diagnostic accuracy and efficiency (Rodrigues et al., 2023).

Machine learning (ML) algorithms have demonstrated impressive capabilities in analyzing complex datasets and extracting meaningful patterns in other diseases such as autism spectrum disorder (ASD) or ADHD (Eslami et al., 2021; Bahathiq et al., 2022; Ehrig et al., 2023). By harnessing the power of computational algorithms, researchers can integrate diverse data sources, including physical and cognitive variables, to develop predictive models for FASD diagnosis (Blanck-Lubarsch et al., 2022; Ehrig et al., 2023). Such models have the potential to augment existing diagnostic frameworks, enabling clinicians to make more informed decisions and speeding up the diagnostic process.

In the present study, supervised classification ML algorithms were employed to construct a predictive diagnosis model of FASD and its subtypes. The model was trained using sociodemographic, clinical and psychological variables. ML provides a powerful tool for prediction and feature importance determination, especially when data patterns may be too complex for conventional statistical methods. The algorithms investigated include Logistic Regression (LR), Linear Discriminant Analysis (LDA), Support Vector Machine (SVM), K-nearest Neighbors (KNN), Random Forest (RF) and eXtreme Gradient Boosting (XGB).

This study aims to develop ML algorithms that use physical and neurocognitive data from children with FASD. The algorithms will identify a distinctive FAS profile in the dataset to enhance FASD diagnosis compared to the current methods. This research aims to provide more accurate diagnostic tools for the assessment of FASDs, which could revolutionize clinical practice, thereby facilitating the initiation of early therapies and improving the quality of life of people affected by this silent disease.

## 2 Material and methods

### 2.1 Study design and participant information

This is a multicentre and pilot investigation. The study included all patients from the Catalan Institute for Fostering and Adoption (ICAA) database, who agreed to participate. The total study cohort comprised 231 patients, which includes 73 control patients and 158 patients diagnosed with FASD. The study, registered at clinicaltrials.gov (NCT02558933), integrated cohorts from previous investigations (PI13/01135; OG085818; PI16/00566; PI19/01853) that included participants enrolled between March 2017 and November 2023. The study was conducted at the Hospital del Mar Medical Research Institute of Barcelona and Hospital Clinic of Barcelona, and all procedures adhered to ethical standards outlined in the Declaration of Helsinki and Spanish data privacy regulations. Consent was obtained from the caregiver or legal representative of patients due to their incapacity to provide informed consent, as approved by the Comité Ético de Investigación Clínica Parc de Salut MAR (No. HCB/2021/0459).

The minimum sample size calculation was conducted using G^*^Power software (Faul et al., 2007) with the following parameters: bilateral contrast, alpha 0.1, beta cut-off of 0.2, corresponding to a power of 0.8 (Gupta et al., 2016), estimated proportion of replacements required (20%), precision 0.1 (90%) and estimate 50% of the population affected. A minimum of 70 samples in two independent groups (non-FASD and FASD groups or Non-FASD vs. each FASD subtype) were required to compute the necessary sample size.

### 2.2 FASD diagnosis and clinical evaluation

To diagnose FASD, all adopted children in the EEC who were included in this study, including those with verified prenatal alcohol exposure (PAE), underwent independent examination using standardized dysmorphology exams (Hoyme et al., 2016). The diagnostic category for each child was identified based on the 1996 IOM standards (reviewed in 2016) (Hoyme et al., 2005, 2016), which consist of five diagnostic characteristics. (1) confirmed prenatal alcohol exposure; (2) evidence of a characteristic minor facial abnormalities pattern, typified by having a thin upper lip, smooth philtrum and short palpebral fissures; (3) growth retardation, defined as height or weight ≤ 10th percentile; (4) evidence of deficient brain growth or subrogated data; and (5) behavioral or cognitive affected domains (1 or 2) related to prenatal alcohol exposure. For a diagnosis of complete FAS, criteria 2, 3, 4, 5 (confirmed or not confirmed prenatal alcohol exposure) were required. For partial FAS, criteria 1, 2, and at least one of criteria 5 (confirmed prenatal alcohol exposure) or 2, 5 and 3 or 4 (no confirmed prenatal alcohol exposure) were required. The diagnosis of alcohol-related birth defects (ARBD) required the finding of one criterion plus a minimum of one structural defect involving heart, skeleton, kidney, eye, ear or minor abnormalities like railway ears, midface hypoplasia or stick hockey hands. The diagnosis of alcohol-related neurodevelopmental disorders (ARND) required the finding of 1 and 5 criteria.

The variables selected for our study were based on IOM classification from 2016 (Hoyme et al., 2016) to FASD diagnosis. Selected sociodemographic variables are related to maternal alcohol consumption during pregnancy (Hoyme et al., 2016), origin and ethnicity (Oh et al., 2023a). Parent feeling variables were included in our selection as they may be relevant to determine if parental perceptions play a role in the diagnosis of FASD predicted by ML algorithms. For clinical variables were selected growth deficits (Astley et al., 2016; Hoyme et al., 2016; Treit et al., 2016), craniofacial dysmorphology (Smith et al., 2014; Hoyme et al., 2016), birth malformations (Dylag et al., 2023), neurodevelopmental disorders (Geier and Geier, 2022) and other physical features and medical history (Brennan and Giles, 2014; del Campo and Jones, 2017; Ninh et al., 2019). Lastly, related to neuropsychological domains, we selected variables significant for FASD diagnosis, including motor cognition (Bakoyiannis et al., 2014), language (Hendricks et al., 2019), academic achievement (Glass et al., 2017), memory (Rasmussen, 2005), attention (Young et al., 2016), executive functioning including impulse control and hyperactivity (Peadon and Elliott, 2010), affect regulation (Temple et al., 2019) and adaptive behavior, social skills, or social communication (Temple et al., 2019; Hammond et al., 2022).

### 2.3 Neurocognitive assessment

Cognitive assessment utilized the Wechsler Intelligence Scale for Children (WISC) series, using the Fifth Edition (WISC-V) (Weiss et al., 2019). Evaluating full-scale Intelligence Quotient (IQ), Verbal Comprehension (VCI), Visuospatial Index (VSI), Perceptual Reasoning (FRI), Working Memory (WMI), and Processing Speed (PSI).

Adults' cognitive functioning was evaluated using the Wechsler Adult Intelligence Scale (WAIS), the Fourth Edition (WAIS-IV) (Wechsler, 2008). Preschoolers' cognitive abilities were assessed using the Wechsler Preschool and Primary Scale of Intelligence (WPPSI-IV) (Raiford and Coalson, 2014). Additionally, the Adult Self-Report (ASR/18-59) (Achenbach and Rescorla, 2003) collected self-reported data on behavioral concerns in adults, while the Child Behavior Checklist (CBCL) (Achenbach, 2004) gathered parental reports on children aged 6–18. All assessments adhered to unified criteria and professionals received standardized training, ensuring consistency and reliability. Data were recorded in a confidential database, maintaining accuracy and confidentiality throughout the research process.

### 2.4 Statistical analysis

Statistical analysis was performed using SPSSv22 and R. Graphs were performed using Graphpad Prism 8.0 software. Descriptive analysis was used to characterize the samples. Categorical variables were presented as counts and percentages, while continuous variables were presented as means and standard deviations. Relationships between sociodemographic, clinical and neuropsychological features were examined for quantitative factors using Kruskall–Wallis test with Dunn's correction for multiple comparisons and for qualitative variables chi-square test. A significance level of *p* < 0.05 was applied to all analyses.

In addition to the aforementioned statistical tests, machine learning (ML) algorithms were also employed to create a predictive model, using the statistical software R (3.3.0+ version).

### 2.5 Machine learning models

This study employed several ML algorithms to predict FASD and its subtypes (FAS, pFAS and ARND), such as LR, LDA, linear SVM, polynomial SVM, KNN, RF and XGB (Zhang et al., 2019). The data underwent a preparation phase, where “mice” function from *VIM* package was used for missing values imputation, employing the predictive mean matching method (pmm) (Kowarik and Templ, 2016). This process was repeated 5 times, as per default setting. Just 1% of the data were missing and single imputation is considered appropriate when <5% of the data are missing (Graham, 2009). The dataset was subsequently scaled using “scale” function in base R.

LR is a binary classification algorithm, which uses a logistic function to predict class probability. Coefficients are obtained using maximum likelihood estimation (Hosmer et al., 2013). LR is easy to implement and performs well on linearly separable classes. However, it may overfit with many features and struggles with complex relationships. LDA projects data into a lower-dimensional space, maximizing class separability and minimizing variance within a class, finding a linear combination of features that characterizes a group (Gardner-Lubbe, 2021). SVM maximizes the distance between the separating hyperplane of the variables to classify (Huang et al., 2018). In our study, linear SVM and polynomial SVM are differentiated. Linear SVM classifies linearly separable data and is more computationally efficient. Polynomial SVM classifies non-linearly separable data, transforms input space into a higher-dimensional space, finds more complex relationships, and is computationally intense (López et al., 2022). KNN predicts class by calculating the Euclidean distance to all training points and selecting K most similar instances (the neighbors). It handles multiclass classification and learns complex decision boundaries (Zhang, 2016). However, it performs poorly on high-dimensional datasets because the distance to all neighbors must be recalculated. Ensemble methods like RF and XGB are decision tree-based algorithms. RF combines multiple independently trained decision trees, uses bagging to create subsets of the original dataset, and then aggregates the results (Denisko and Hoffman, 2018). On the other hand, XGB trains decision trees sequentially, with each new tree correcting errors made by the previous one (Li et al., 2022). The ML algorithms used in this study has its own strengths and weaknesses, leading to varied results. The range of ML algorithms compared spans from traditional predictive models like LR to more complex ensemble methods like RF and XGB, which are capable of handling high-dimensional data. By comparing different models, our study aimed to find the most effective model for predicting FASD and its subtypes. This diversity in approaches enhances the robustness and comprehensiveness of the study.

For the analysis, a total of 66 variables were selected, encompassing five sociodemographic parameters, 35 clinical features, six intelligence scores and 20 behavioral domains (Tables 1–4). Prior to model construction, a hold-out method was applied to split the data into training and test sets using “createDataPartition” function from *caret* package in R (Kuhn, 2008). Sixty-seven percent of the data was allocated to training set and the remaining 33% to test set. This function employs a stratified random sampling method, which minimizes the bias of the data distribution and creates balanced data.

**Table 1:** Sociodemographic data of children population (*n* = 231).

	**Non-FASD**		**FAS**		**pFAS**		**ARND**		***p-*value**
	***n*** = **73**	**%**	***n*** = **33**	**%**	***n*** = **81**	**%**	***n*** = **44**	**%**	
**Sex**									0.105
Male	53	72.6	16	48.48	51	62.96	30	68.18	
Female	20	27.4	17	51.52	30	37.04	14	31.82	
**Age**									0.161
<6	2	2.74	0	0.00	4	4.59	0	0.00	
7–16	51	69.86	24	72.73	47	58.0	22	50.00	
<17	20	27.4	9	27.27	30	37.0	22	50.00	
**Origin**									0.162
Russia	51	69.86	24	72.73	53	65.43	23	52.27	
Spain	9	12.33	1	3.03	9	11.11	10	22.73	
Ucrania	9	12.33	4	12.12	12	14.81	4	9.09	
Colombia	1	1.37	1	3.03	1	1.23	2	4.55	
Poland	0	0	1	3.03	2	2.47	1	2.27	
Argentina	0	0	1	3.03	0	0.00	1	2.27	
Bolivia	0	0	0	0.00	1	1.23	0	0.00	
Camboya	0	0	1	3.03	0	0.00	0	0.00	
England	0	0	0	0.00	0	0.00	2	4.55	
Hungria	0	0	0	0.00	1	1.23	0	0.00	
Kazajastan	0	0	0	0.00	2	2.47	0	0.00	
Mexico	0	0	0	0.00	0	0.00	1	2.27	
Morraco	1	1.37	0	0.00	0	0.00	0	0.00	
Rumania	2	2.74	0	0.00	0	0.00	0	0.00	
**Ethnicity**									0.15
Caucasic	71	97.26	30	90.91	77	95.06	40	90.91	
Latinamerican	1	1.37	2	6.06	2	2.47	4	9.09	
Kazakh	0	0	0	0.00	2	2.47	0	0.00	
Arab	1	1.37	0	0.00	0	0.00	0	0.00	
Asiatic	0	0	1	3.03	0	0.00	0	0.00	
**Parents feeling**									
Aggressive	34	46.58	4	9.09	34	41.98	29	65.91	0.06
Emotion regulation	42	57.53	13	39.39	59	72.84	25	56.82	0.16
Depression	38	52.05	22	66.67	43	53.09	35	79.55	0.06
Low self-steem	42	57.53	21	63.64	33	40.74	8	18.18	0.09
Motivation	48	65.75	10	30.30	50	61.73	21	47.73	0.20

Chi-square test was used to compare the outcomes of the different groups: non-FASD, FAS, pFAS and ARND. Significant differences were considered when p-value <0.05. ARND, alcohol-related neurodevelopmental disorder; FASD, fetal alcohol spectrum disorders; FAS, fetal alcohol syndrome; pFAS, partial fetal alcohol syndrome.

In addition to the hold-out method, a resampling method involving four-fold cross-validation and three repeats was adopted. This was implemented using “trainControl” function from the *caret* package (Kuhn, 2008). The models were trained using “train” function with hyperparameters set to default, which gathers and simplifies numerous R algorithms for the development of predictive models (Kuhn, 2008). The models employed included LR, using “glm” method and binomial family, and LDA, implemented with “lda” method, which has “moment” as the default mean and variance estimator. Linear SVM and Polynomial SVM were performed using “svmLinear” and “svmPoly” methods, respectively. They have C tuning parameter, which determines the margin classification, equal to 1 as default settings. KNN was employed by “knn” method also from *caret* package, performing automatic hyperparameter tuning for k depending on instance-based learning. In addition, RF was employed using “rf” method, with 500 trees as default. XGB model used “xgbTree” method, having 100 maximum iterations by default.

The “predict” function from *stats* package was used to predict classes with the test group. In order to make comparisons, the “confusionMatrix” function from *caret* package was used to calculate true positive, true negative, false positive and false negative. These calculations provided measures including accuracy, precision, sensitivity, F1 score and specificity. ROC-AUC was obtained using “roc” function from *pROC* package (Robin et al., 2011). Training and test datasets were consistent across FASD and its subgroups, ensuring a fair and valid comparison.

Feature importance prediction of the models was determined by calculating the Root Mean Square Error (RMSE) loss after permutation. It was obtained with “explain” function from *DALEX* package, with “classification” type model in arguments (Law Biecek, 2018). Plots were generated from the object class formed by “variable_importance” function from *caret* package (Kuhn, 2008).

## 3 Results

### 3.1 FASD profile

**Figure 1:**
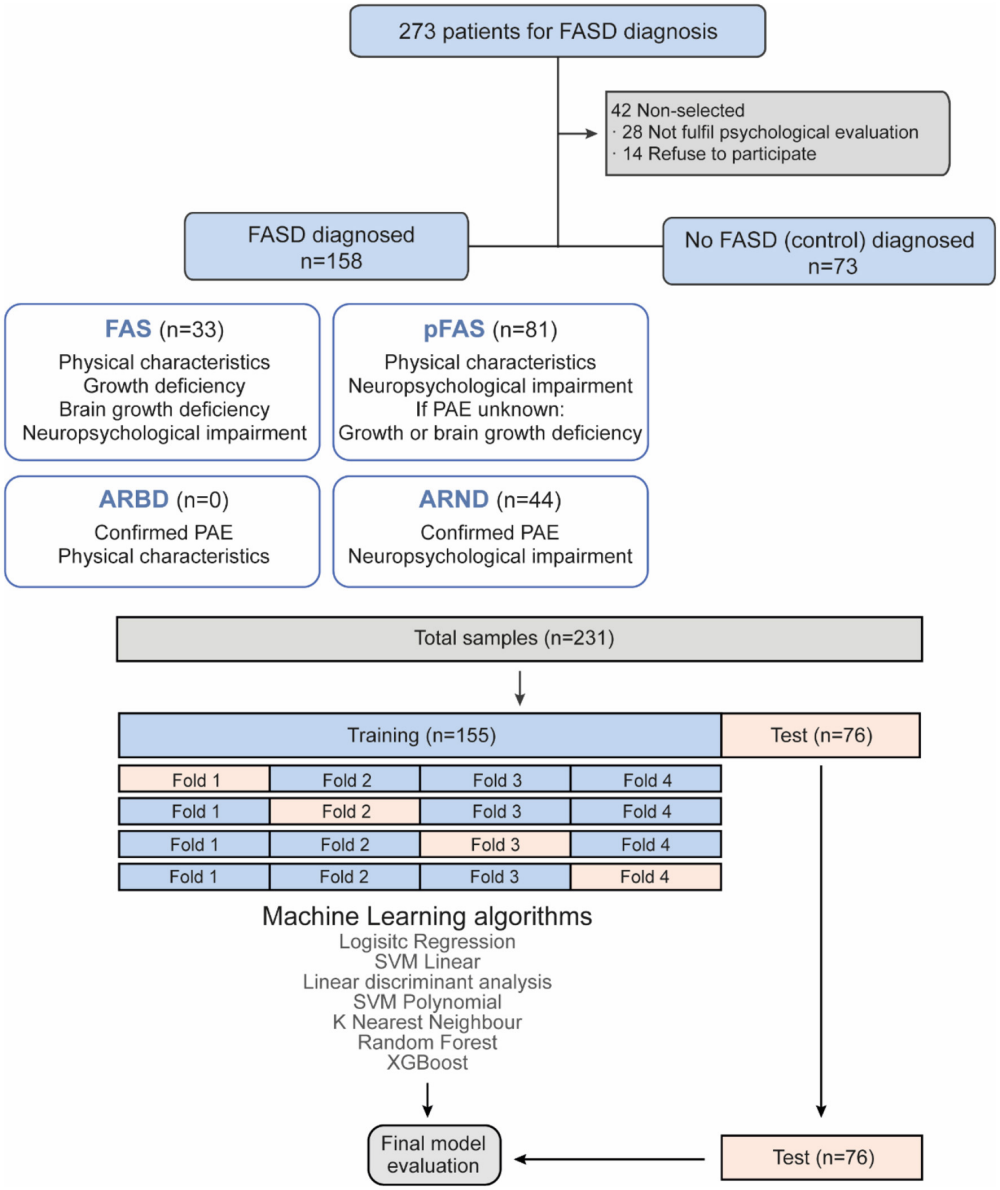
Study flowchart. Flowchart of FASD and non-FASD diagnosis and machine learning prediction.

The study initially included 273 patients. However, 42 were excluded: 28 lacked psychological evaluations and 14 refused participation. Of the remaining 231 subjects, 73 were diagnosed as non-FASD (controls), and 158 were diagnosed with FASD, comprising 33 with FAS, 81 with pFAS, and 44 with ARND. A database was compiled with sociodemographic and psychological characteristics of both FASD patients (and their subtypes) and non-FASD participants (Figure 1).

Sociodemographic characteristics of the population were collected from FASD and non-FASD patients (Table 1). The chi-square test revealed no significant differences between groups. Significant differences were found in physical characteristics between children diagnosed with FASD and non-FASD (Table 2, Supplementary Table 1). Prematurity (*p*-value = 0.011) was higher in FAS children compared to non-FASD children (*p*-value = 0.018; Supplementary Table 1). Growth retardation (*p* < 0.001) was also higher in FAS and ARND children compared to non-FASD children (*p*-value <0.001; *p*-value = 0.040). Birth complications (*p*-value = 0.047), as perinatal asphyxia or abnormal heart rate, were more prevalent in ARND children compared to non-FASD children (*p*-value = 0.052). As expected, maternal alcohol consumption confirmation also showed significant differences (*p*-value <0.001) in all groups compared to non-FASD patients.

**Table 2:** Clinical and physical features of children population (*n* = 231).

	**Non-FASD**		**FAS**		**pFAS**		**ARND**		***p-*value**
	***n*** = **73**	**%**	***n*** = **33**	**%**	***n*** = **81**	**%**	***n*** = **44**	**%**	
ADHD	39	53.42	17	51.52	47	58.02	22	50.00	0.825
Autism	4	5.48	3	9.09	3	3.70	0	0.00	0.25
CNS problems	12	16.44	10	30.30	18	22.22	6	13.64	0.246
Corpus Callosum affectation	1	1.37	2	6.06	2	2.47	0	0.00	0.309
Epilepsy	0	0	1	3.03	1	1.23	0	0.00	0.399
Premature	10	13.7	12	36.36	18	22.22	4	9.09	**0.011**
Growth retardation	6	8.22	14	42.42	14	17.28	10	22.73	**<0.001**
Birth diseases	19	26.03	16	48.48	33	40.74	21	47.73	**0.047**
Maternal alcohol consumption confirmation	9	12.33	19	57.58	51	62.96	44	100.00	**<0.001**
Maternal tobacco consumption confirmation	1	1.37	4	12.12	8	9.88	4	9.09	0.117
Maternal illicit drug use	3	4.11	2	6.06	3	3.70	4	9.09	0.584
Patient licit drug use	16	21.92	3	9.09	16	19.75	13	29.55	0.179
Patient illicit drug use	12	16.44	3	9.09	10	12.35	8	18.18	0.615
Height affectation	3	4.11	27	81.82	13	16.05	11	25.00	**<0.001**
Weight affectation	3	4.11	24	72.73	18	22.22	6	13.64	**<0.001**
Microcephaly	10	13.7	29	87.88	38	46.91	8	18.18	**<0.001**
Short palpebral fissures	21	28.77	23	69.70	44	54.32	11	25.00	**<0.001**
Lip-philtrum affectation	31	42.47	33	100.00	80	98.77	10	22.73	**<0.001**
Face morphology affectation	3	4.11	5	15.15	15	18.52	3	6.82	**0.025**
Ears affectation	10	13.7	9	27.27	15	18.52	7	15.91	0.391
Eyes affectation	19	26.03	17	51.52	26	32.10	8	18.18	**0.013**
Nose affectation	4	5.48	4	12.12	12	14.81	1	2.27	0.066
Mouth prognathism	6	8.22	4	12.12	5	6.17	2	4.55	0.603
Neck affectation	0	0	0	0.00	0	0.00	1	2.27	0.234
Chest affectation	0	0	0	0.00	1	1.23	0	0.00	0.602
Cardiac affectation	2	2.74	6	18.18	7	8.64	5	11.36	0.06
Abdomen affectation	0	0	1	3.03	1	1.23	0	0.00	0.399
Back affectation	1	1.37	0	0.00	3	3.70	0	0.00	0.353
Pelvic affectation	1	1.37	2	6.06	4	4.94	0	0.00	0.41
Upper limbs affectation	1	1.37	7	21.21	6	7.41	1	2.27	**0.001**
Hands affectation	18	24.66	9	27.27	28	34.57	7	15.91	0.15
Lower limbs affectation	0	0	0	0.00	1	1.23	3	6.82	**0.034**
Feet affectation	1	1.37	1	3.03	3	3.70	3	6.82	0.481
Renal affectation	2	2.74	1	3.03	0	0.00	1	2.27	0.519
Anemia	2	2.74	1	3.03	6	7.41	4	9.09	0.385

Chi-square test was used to compare the outcomes of the different groups: non-FASD, FAS, pFAS and ARND. Significant differences are bold and were considered when p-value <0.05. ARND, alcohol-related neurodevelopmental disorder; ADHD, attention-deficit/hyperactivity disorder; CNS, central nervous system; FASD, fetal alcohol spectrum disorders; FAS, fetal alcohol syndrome; pFAS, partial fetal alcohol syndrome.

FASD patients showed lower height (*p* < 0.0001) by 81%, 16%, and 25% for FAS, pFAS, and ARND respectively, compared to non-FASD (Table 2, Supplementary Table 1). Weight alterations (*p* < 0.0001) increased significantly in FAS and pFAS. Microcephaly (*p*-value <0.0001), shorter palpebral fissures (*p*-value = 0.01) and lip-philtrum affectation (*p*-value <0.001) were more prevalent in FAS and pFAS. Facial anomalies (*p*-value = 0.025) were significantly higher in pFAS (*p*-value = 0.009) compared to non-FASD group. In particular, children with affected eyes (*p*-value = 0.013) and upper limbs (*p* = 0.001) were predominantly from FAS groups compared to non-FASD (*p*-value = 0.037 and *p*-value = 0.001, respectively). Moreover, significant disparities in lower limbs (*p*-value = 0.034) were observed, primarily in ARND compared to non-FASD group.

Among FASD groups (Table 2, Supplementary Table 1), significant differences included prematurity (*p*-value = 0.009), eyes (*p*-value = 0.011) and upper limbs affectation (*p*-value = 0.011) between FAS and ARND. FAS exhibited higher growth retardation levels compared to pFAS (*p*-value = 0.014). Maternal alcohol consumption, short palpebral fissures and lip-philtrum affectation showed increased levels in FAS (*p*-value = 0.042, *p*-value = 0.003 and *p*-value <0.001, respectively) and pFAS patients (*p*-value = 0.023, *p*-value = 0.018 and *p*-value <0.001, respectively) compared to ARND, confirming that this group does not exhibit physical characteristics. Microcephaly varied among all FASD groups, showing 87% of the cases in FAS, 46% in pFAS and 18% in ARND. No significant differences were noted in other physical or clinical characteristics, except for a trend toward greater cardiac damage in FASD (*p*-value = 0.06).

Significant differences were observed in psychological intelligence parameters between FASD and non-FASD patients. Evaluating cognitive performance with WISC V, children diagnosed with FASD, specifically FAS and pFAS groups (Table 3, Supplementary Table 2), exhibited lower scores on VCI (*p*-value = 0.001), VSI (*p*-value = 0.005), FRI (*p*-value = 0.001), WMI (*p*-value <0.001), PSI (*p*-value = 0.003) and IQ (*p*-value <0.001). No significant differences were found for the WAIS-IV and WPPSI-IV tests.

**Table 3:** Intelligence scale data of children population (*n* = 231).

	**Non-FASD**		**FAS**		**pFAS**		**ARND**		***p-*value**
	**Mean**	**SD**	**Mean**	**SD**	**Mean**	**SD**	**Mean**	**SD**	
**WAIS-IV**									
Verbal Comprehension Index (VCI)	36.17	14.53	38.25	7.54	39.67	7.40	37.91	11.81	0.748
Perceptual Reasoning Index (PRI)	32.11	10.15	36.50	8.70	34.56	7.52	38.00	12.39	0.698
Working Memory Index (WMI)	34.43	7.04	27.50	8.10	32.90	10.47	40.00	12.69	0.264
Processing Speed Index (PSI)	33.44	9.42	30.50	9.33	34.22	10.54	35.89	9.33	0.839
Intelligence Quotient (IQ)	68.79	8.08	72.33	14.73	64.58	10.27	66.68	6.63	0.453
**WISC-V**									
Verbal Comprehension Index (VCI)	82.05	13.67	69.67	11.52	76.34	11.32	80.40	12.29	**0.001**
Visuospatial Index (VSI)	94.28	17.30	83.77	13.93	84.77	14.37	94.32	15.13	**0.005**
Fluid Reasoning Index (FRI)	89.96	13.60	81.44	14.60	82.47	14.21	93.12	12.83	**0.001**
Working Memory Index (WMI)	91.16	15.94	74.92	11.94	78.64	14.08	88.08	14.33	**0.000**
Processing Speed Index (PSI)	91.19	16.75	80.48	12.81	82.49	16.39	90.16	12.45	**0.003**
Intelligence Quotient (IQ)	84.91	14.31	70.04	10.27	73.68	11.69	80.74	9.81	**0.000**
**WPPSI-IV**									
Intelligence Quotient (IQ)	76.50	12.02	0.00	0.00	83.50	2.12	0.00	0.00	0.741
Verbal Comprehension Index (VCI)	82.00	9.90	0.00	0.00	91.00	8.49	0.00	0.00	0.15
Visuospatial Index (VSI)	79.00	0.00	0.00	0.00	104.50	2.12	0.00	0.00	0.191
Fluid Reasoning Index (FRI)	97.00	4.24	0.00	0.00	102.00	33.94	0.00	0.00	0.223
Working Memory Index (WMI)	62.00	15.56	0.00	0.00	94.00	12.73	0.00	0.00	0.819
Processing Speed Index (PSI)	79.50	24.75	0.00	0.00	88.50	13.44	0.00	0.00	0.819

Kruskal-Wallis test was used to compare the outcomes of the different groups: non-FASD, FAS, pFAS and ARND. Significant differences are bold and were considered when p-value <0.05.

ARND, alcohol-related neurodevelopmental disorder; FASD, fetal alcohol spectrum disorders; FAS, fetal alcohol syndrome; pFAS, partial fetal alcohol syndrome; SD, standard deviation.

Related to behavioral parameters, CBCL 6–18 test showed impairments in several cognitive domains in FASD patients compared to their non-FASD counterparts (Table 4, Supplementary Table 3). Increased levels of thought problems (*p*-value = 0.035), rule breaking behavior (*p*-value = 0.002), externalizing problems (*p*-value = 0.045), total problems (*p*-value = 0.008), anxiety problems (*p*-value = 0.009), obsessive compulsive problems (OCP; *p*-value = 0.049) and stress problems (*p*-value = 0.001) domains were observed in ARND compared to non-FASD. Significantly increased levels of attention problems were observed in FAS (*p*-value = 0.021) and ARND (*p*-value = 0.020) compared to non-FASD. Within the FASD group found significant differences in thought problems (*p*-value = 0.007), anxiety problems (*p*-value = 0.006) and OCP (*p*-value = 0.003), with significantly increased levels in the ARND group compared to pFAS. Furthermore, ARND showed high levels of rule-breaking behavior compared to FAS and pFAS subgroups (*p*-value = 0.002 and *p*-value = 0.002), externalizing problems (*p*-value = 0.003 and *p*-value = 0.002), total problems (*p*-value = 0.006 and *p*-value = 0.021), oppositional defiant problems (*p*-value = 0.002 and *p*-value = 0.018), conduct problems (*p*-value = 0.016 and *p*-value = 0.009) and stress problems (*p*-value <0.001 and *p*-value = 0.015), respectively. Moreover, the results display significantly increased levels of aggressive behavior (*p*-value = 0.01) in the ARND group compared to FAS.

**Table 4:** Behavior data of children population (*n* = 231).

	**NOFAS**		**FAS**		**pFAS**		**ARND**		***p-*value**
**Mean**	**SD**	**Mean**	**SD**	**Mean**	**SD**	**Mean**	**SD**	
**CBCL**									
Anxious or depressed	64.85	10.73	65.25	9.65	63.84	10.68	69.35	10.12	0.084
Withdrawn depressed	61.99	11.23	64.36	12.09	60.49	9.93	65.23	12.00	0.192
Somatic complaints	58.99	9.10	57.43	7.38	59.00	7.86	62.74	9.33	0.083
Social problems	66.91	11.74	69.18	8.95	67.87	8.70	70.94	8.77	0.118
Thought problems	65.18	10.27	66.86	9.23	63.83	9.18	69.39	7.13	**0.048**
Attention problems	69.27	12.24	73.82	9.88	70.76	8.60	73.58	11.58	**0.042**
Rule breaking behavior	62.82	9.93	60.07	8.30	61.06	8.59	67.10	7.94	**0.007**
Aggressive behavior	67.97	13.04	63.61	11.42	64.02	11.28	70.68	10.76	**0.015**
Internalizing problems	62.81	10.02	63.64	9.48	61.21	12.40	67.73	9.09	0.052
Externalizing problems	64.95	11.84	61.32	10.35	62.54	10.18	68.97	8.30	**0.007**
Total problems	67.37	10.07	67.68	6.96	67.25	7.71	71.87	7.12	**0.027**
Depressive problems	64.54	10.80	63.43	9.72	64.11	9.28	67.26	8.72	0.262
Anxiety problems	66.07	11.30	67.18	10.13	65.44	10.40	71.87	10.56	**0.033**
Somatic problems	57.25	7.93	54.96	7.08	56.81	6.92	59.35	9.48	0.246
Attention deficit	65.96	8.43	67.43	6.73	65.95	7.07	68.26	8.00	0.435
Oppositional defiant problems	64.72	9.94	62.39	9.08	61.75	9.16	67.94	8.94	**0.014**
Conduct problems	63.72	10.80	61.04	9.67	62.21	9.44	66.77	8.32	**0.045**
Sluggish cognitive tempo	60.98	8.26	63.07	8.57	62.66	7.55	65.70	8.81	0.129
Obsessive compulsive problems	62.63	11.48	62.71	10.34	60.30	10.61	67.37	11.42	**0.034**
Stress problems	67.39	10.73	67.71	7.89	65.25	9.48	74.53	10.27	**0.001**
**ASR/18-59**									
Anxious or depressed	72.67	9.14	69.60	14.67	65.11	11.06	65.23	6.61	0.414
Withdrawn depressed	69.00	9.21	62.20	4.27	65.89	10.75	71.85	9.54	0.16
Somatic complaints	65.50	13.94	54.60	3.71	62.06	8.11	62.31	9.57	0.274
Thought problems	71.67	9.61	65.40	6.19	63.00	9.11	63.85	9.09	0.351
Attention problems	72.00	9.47	68.40	13.26	66.28	8.76	76.62	8.47	**0.032**
Rule breaking behavior	73.67	9.27	63.20	9.98	64.67	8.30	70.62	10.86	0.108
Aggressive behavior	70.33	12.11	61.40	6.80	64.94	9.95	65.62	10.05	0.564
Internalizing problems	72.17	9.20	65.40	6.50	65.63	12.18	67.85	5.68	0.508
Externalizing problems	71.83	8.98	62.00	7.71	64.88	10.47	68.46	9.83	0.328
Total problems	75.00	7.59	66.00	7.31	66.69	10.91	74.62	13.47	0.137
Depressive problems	74.17	12.70	63.80	8.53	67.78	11.00	71.38	7.43	0.171
Anxiety problems	71.50	5.32	69.40	15.08	61.94	7.15	62.31	8.16	0.053
Somatic problems	64.00	13.40	53.40	4.45	61.17	8.15	60.38	11.42	0.366
Sluggish cognitive tempo	67.17	11.69	63.00	7.11	64.19	9.71	67.46	5.49	0.627
Obsessive Compulsive problems	76.17	8.18	65.20	13.42	61.88	11.09	61.77	9.35	0.07

Kruskal–Wallis test was used to compare the outcomes of the different groups: non-FASD, FAS, pFAS and ARND. Significant differences are bold and were considered when p-value <0.05. ARND, alcohol-related neurodevelopmental disorder; FASD, fetal alcohol spectrum disorders; FAS, fetal alcohol syndrome; pFAS, partial fetal alcohol syndrome; SD, standard deviation.

Finally, in the adult behavioral test ASR 18–59 (Table 4, Supplementary Table 3), significant differences related to attention problems (*p*-value = 0.032) were observed, showing ARND higher levels compared to the pFAS subgroup (*p*-value = 0.003).

### 3.2 Machine learning predictive modeling

Predictive models for FASD diagnosis were developed using ML, considering the sociodemographic, clinical, and psychological variables previously discussed. The dataset consisted of 231 samples, with 155 samples used for model training and the remaining 76 samples saved for testing and final model evaluation. A variety of ML algorithms were employed, including XGB, LR, LSVML, LDA, SVMP, kNN, RF and XGB. These models were trained using four-fold cross-validation on the training dataset.

**Figure 2:**
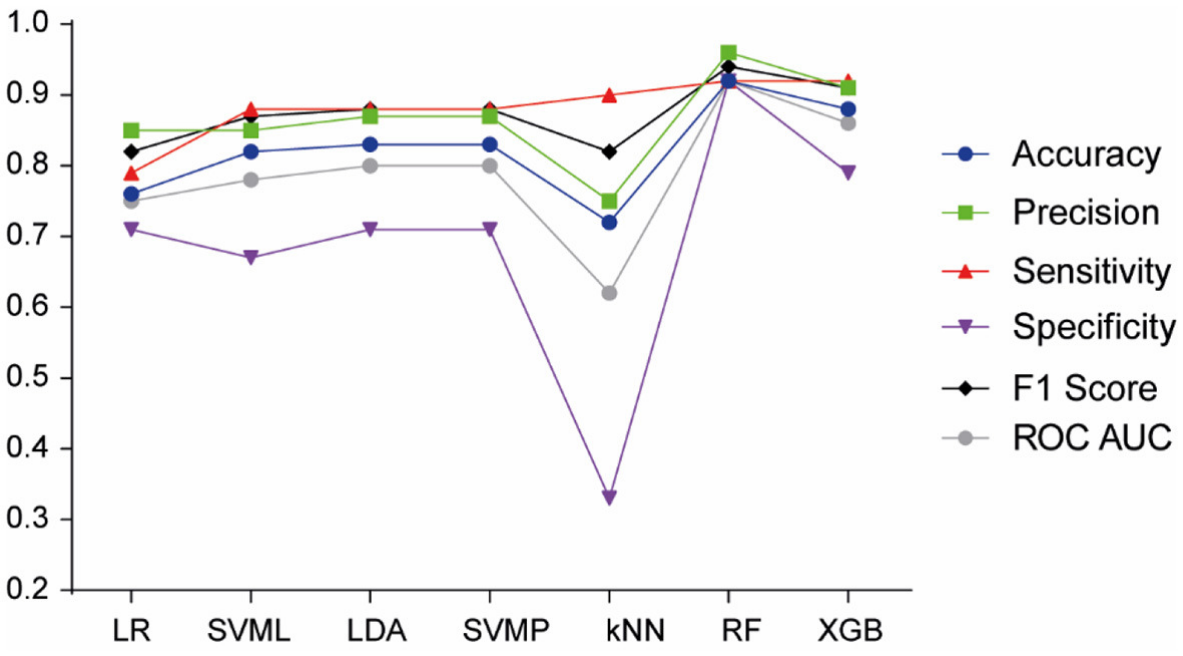
Model performance of machine learning algorithms for FASD prediction. LR, Logistic Regression; SVML, Support Vector Machine Linear Kernel; LDA, Linear Discriminant Analysis; SVMP, Support Vector Machine Polynomial Kernel; KNN, k-Nearest Neighbor; RF, Random Forest; XGB, Gradient-Boosted Trees.

Figure 2 shows the key performance metrics associated with the predictive power of each model. Among the models, the ensemble algorithms (RF and XGB) outperformed the others. Notably, the RF model achieved the highest accuracy (0.92), precision (0.96), sensitivity (0.92), F1 score (0.94), specificity (0.92), and AUC (0.92), establishing it as the most effective model for predicting FASD diagnosis. Other models such as LR, LDA, SVMP, and kNN showed lower performance on these metrics (Figure 2). Consequently, we selected the RF model for our prediction tasks due to its superior discriminative ability.

**Figure 3:**
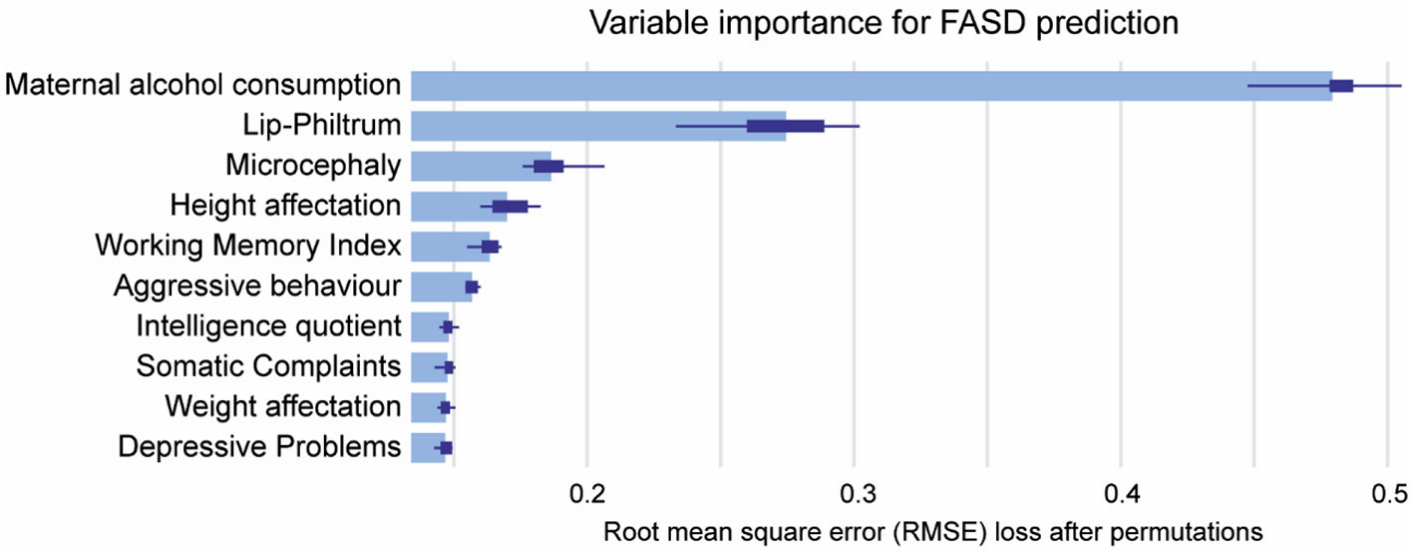
Mean variable-importance of RF model for FASD prediction. Mean variable importance was calculated by using 50 permutations and the root-mean-squared-error-loss-function for the RF model. RF, Random Forest.

To understand the decision-making mechanism of the RF model, we examined the significance of the variables within this algorithm. The features were ranked according to their importance, with maternal alcohol consumption being the most significant (0.48), followed by lip-philtrum (0.27), microcephaly (0.19), height affectation (0.17), Working Memory Index (0.16), aggressive behavior (0.16), Intelligence Quotient (0.15), somatic complaints (0.15), weight affectation (0.15), and depressive problems (0.15; Figure 3). These findings offer crucial insights into the primary attributes associated with FASD conditions and their respective significance in the predictive model.

Another aim of our study is to construct individualized models for each category of FASD. This methodology will allow us to uncover distinct attributes and trends that might remain concealed when all FASD types are examined collectively.

**Figure 4:**
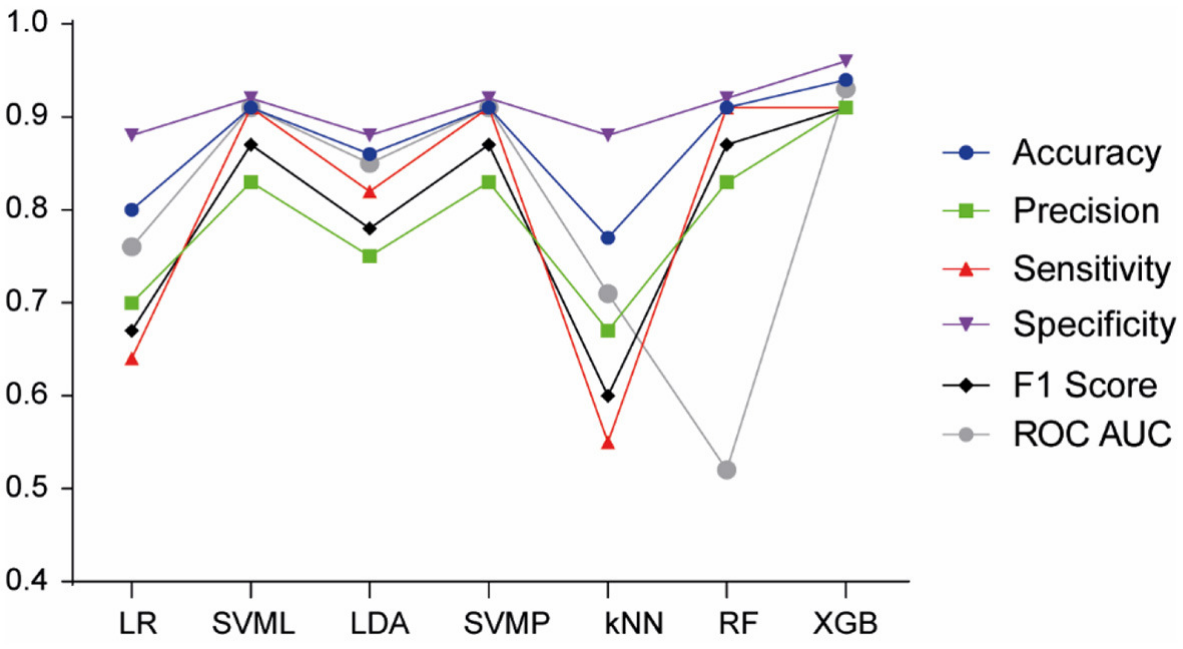
Model performance of machine learning algorithms for FAS prediction. LR, Logistic Regression; SVML, Support Vector Machine Linear Kernel; LDA, Linear Discriminant Analysis; SVMP, Support Vector Machine Polynomial Kernel; KNN, k-Nearest Neighbor; RF, Random Forest; XGB, Gradient-Boosted Trees.

Focusing our analysis on FAS prediction in comparison to non-FASD, we employed the previous ML algorithms. The XGB model outperformed the others (Figure 4), achieving the highest accuracy (0.94), precision (0.91), sensitivity (0.91), F1 Score (0.91), specificity (0.96), and AUC (0.93), thereby proving to be the most effective model for FAS diagnosis prediction.

**Figure 5:**
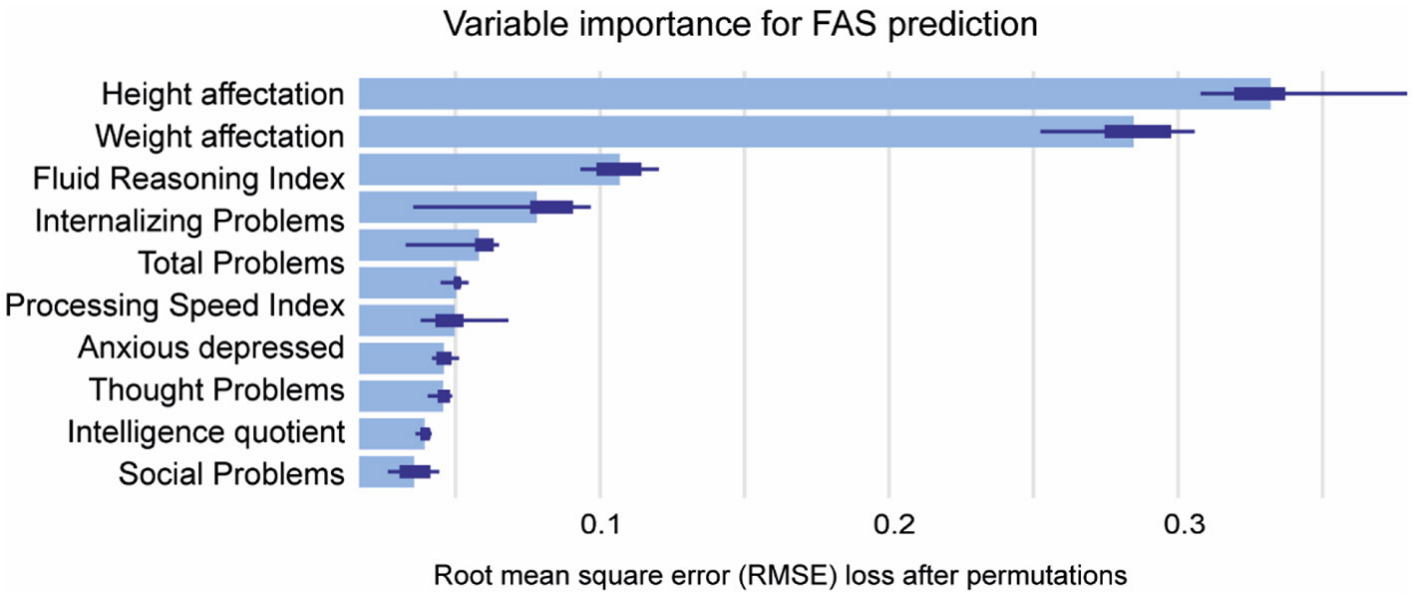
Mean variable-importance of XGB model for FAS prediction. Mean variable importance was calculated by using 50 permutations and the root-mean-squared-error-loss-function for the XGB model. XGB, eXtreme Gradient Boosting.

In Figure 5 the features were ranked based on their importance, with Height (0.32) and Weight (0.28) being the most influential, followed by Fluid Reasoning Index (0.11), Internalizing Problems (0.08), Total Problems (0.1), and Processing Speed Index (0.1) (Figure 5). This highlights FAS prediction is mainly determined by failure to thrive.

**Figure 6:**
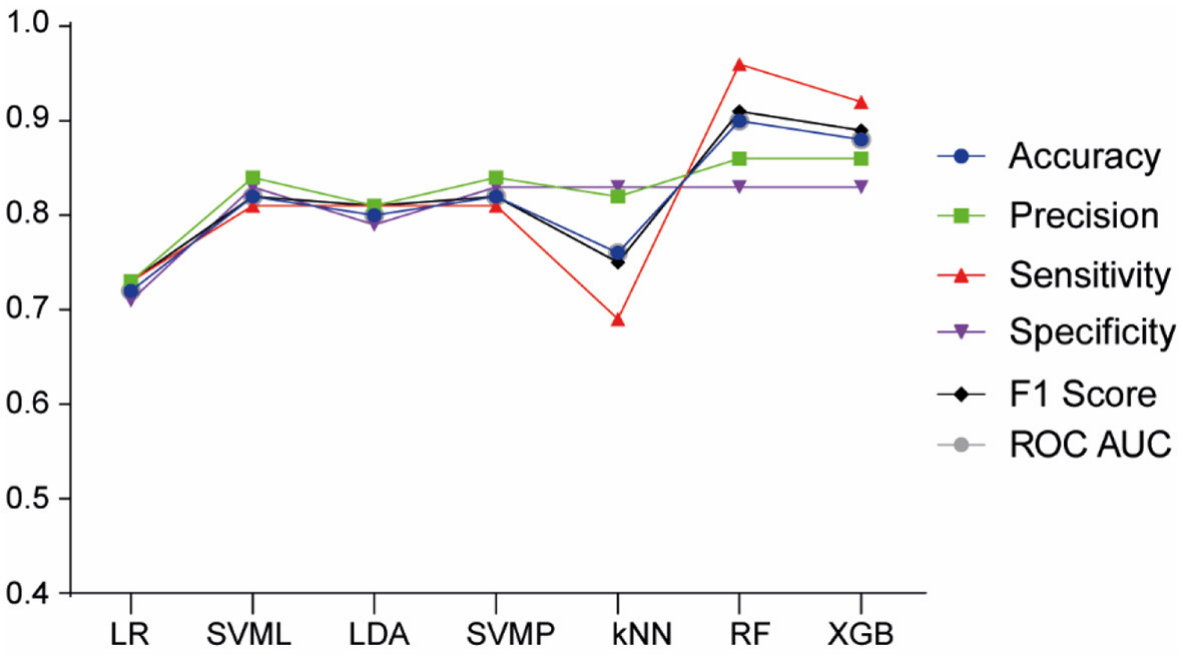
Model performance of machine learning algorithms for pFAS prediction. LR; Logistic Regression; SVML, Support Vector Machine Linear Kernel; LDA, Linear Discriminant Analysis; SVMP, Support Vector Machine Polynomial Kernel; KNN, k-Nearest Neighbor; RF, Random Forest; XGB, Gradient-Boosted Trees.

In the subsequent stage of the research, the focus shifted to the prediction of pFAS compared to non-FASD. Upon evaluating all ML models (Figure 6), the RF model emerged as the most proficient, achieving the highest metrics in accuracy (0.90), precision (0.86), sensitivity (0.96), F1 Score (0.91), specificity (0.83), and AUC (0.90). This underscores its effectiveness in predicting pFAS diagnosis.

**Figure 7:**
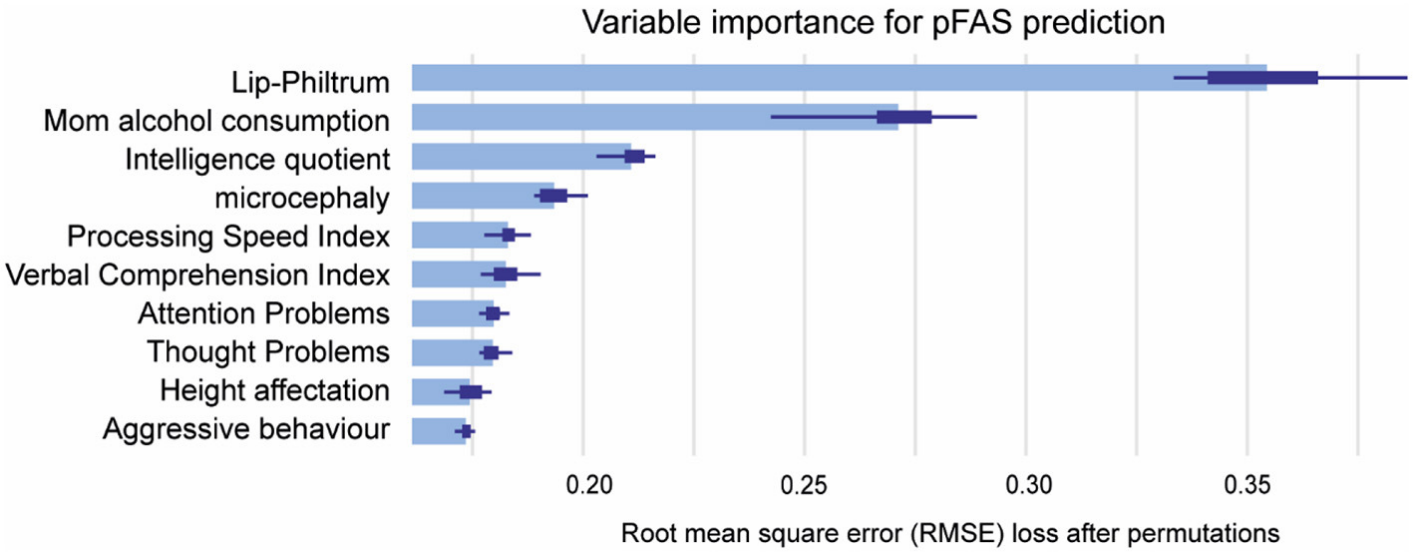
Mean variable-importance of RF model for pFAS prediction. Mean variable importance was calculated by using 50 permutations and the root-mean-squared-error-loss-function for the RF model. RF, Random Forest.

Lip-philtrum (0.36) and Maternal Alcohol Consumption (0.27) were the most impactful features for pFAS prediction, followed by Intelligence Quotient (0.21), Microcephaly (0.18), and Processing Speed Index (0.16), Verbal comprehension index (0.15), attention problems (0.15) and thought problems (0.15; Figure 7).

**Figure 8:**
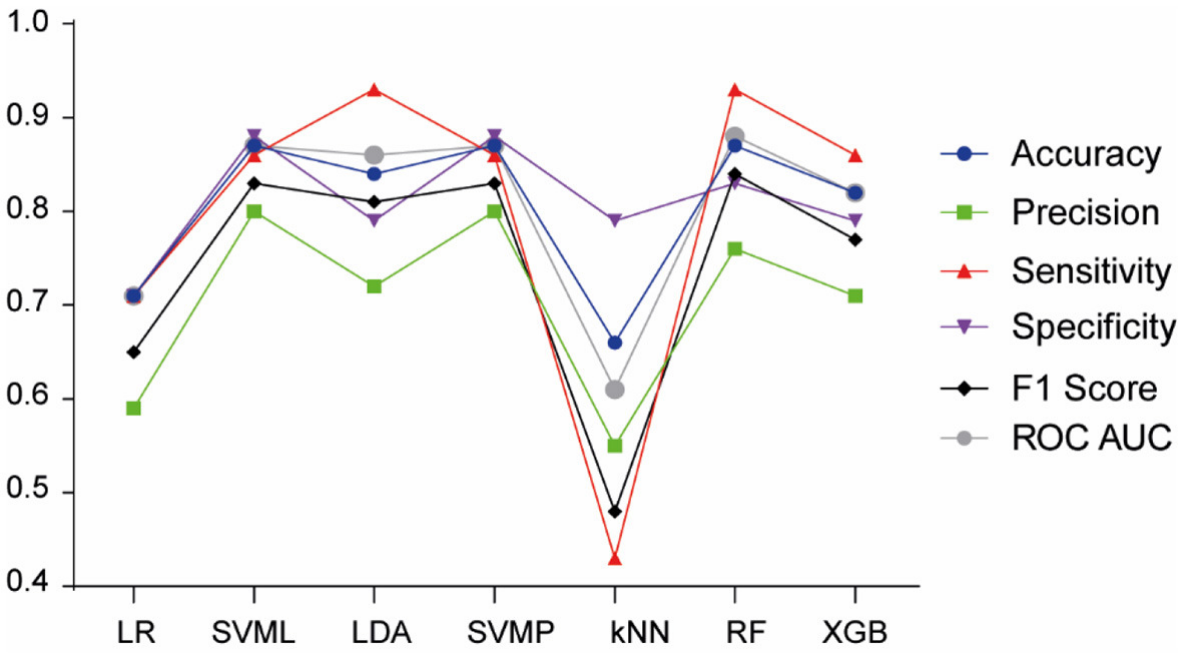
Model performance of machine learning algorithms for ARND prediction. LR, Logistic Regression; SVML, Support Vector Machine linear kernel; LDA, Linear Discriminant Analysis; SVMP, Support Vector Machine polynomial kernel; KNN, k-nearest neighbor; RF, Random Forest; XGB, gradient-boosted trees.

The final phase of the study involved the analysis of ARND prediction. As previously observed, the RF model demonstrated superior performance for ARND prediction (Figure 8), obtaining the best levels of accuracy (0.87), precision (0.76), sensitivity (0.93), F1 Score (0.84), specificity (0.83), and AUC (0.88).

**Figure 9:**
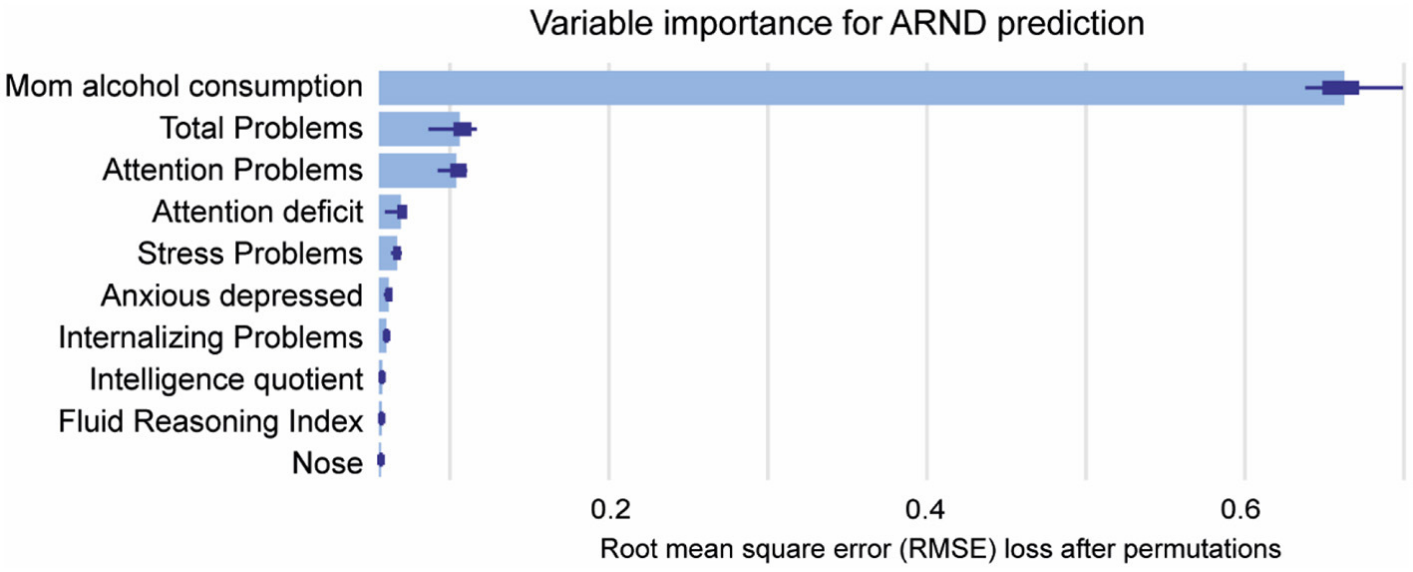
Mean variable-importance of RF model for ARND prediction. Mean variable importance was calculated by using 50 permutations and the root-mean-squared-error-loss-function for the RF model.

Figure 8 displays Maternal Alcohol Consumption (0.64) as the most influential feature for ARND prediction, followed by Total Problems (0.11) and Attention Problems (0.10) (Figure 9). These results confirm the importance of PAE confirmation for diagnosis.

## 4 Discussion

The comprehensive analysis of sociodemographic, clinical, physical, and psychological characteristics in our study provides invaluable insights into the complex nature of FASD and underscores the importance of these features in diagnostic assessment and intervention planning. The variables have been selected based on IOM criteria (Hoyme et al., 2016).

Our study showed that FASD patients share a common profile of maternal alcohol consumption, low height and lip-philtrum affectation (Hoyme et al., 2016). FAS profile shows impaired intelligence domains observed in WISC V test, as previously reported (Bastons-Compta et al., 2016). Prematurity, growth retardation, weight affectation, short palpebral fissures, eyes and upper limbs affectation and attention problems are highlighted in FAS profile compared to non-FASD, being part of the specific diagnosis (Hoyme et al., 2016; Wang et al., 2020). These findings also confirm previous studies from Maschke et al. (2021) that observed facial abnormalities correlate with child's cognitive performance in FRI and WMI in FASD patients. pFAS profile exhibits distinctions from full FAS, particularly in growth problems and physical traits like microcephaly and upper limb impairment, since pFAS does not meet all the requirements of full FAS (Hoyme et al., 2016). ARND profile showed birth diseases, such as perinatal asphyxia or abnormal heart rate and lower limbs affectation. Furthermore, behavioral affectations included thought problems, attention problems, rule-breaking behavior, externalizing, anxiety, obsessive-compulsive and stress problems. ARND lacks certain physical impairments such as weight and height, microcephaly, short philtrum, and eye and upper limb impairments observed in FAS and pFAS (Hoyme et al., 2016). Therefore, these results highlight that FAS and pFAS may need therapies for educational support and intervention for growth retardation. However, ARND group may need a combination of cognitive behavioral therapy, attention training, and psychotherapy for the range of psychological and behavioral problems.

FASD, shares similarities with others neurocognitive disorders as Autism and ADHD, but is distinguished by its association with PAE, causing a distinct pattern of neurodevelopmental impairments (Rommelse et al., 2010; May and Gossage, 2011). Autism is characterized by elevated FRI and VSI, while ADHD is linked to deficiencies in FRI (Tamm and Juranek, 2012; Happé, 2021). Studies of ASD observed that VCI correlated negatively with communication symptoms, and WMI correlated positively with social symptoms (Rabiee et al., 2019). Similarly, individuals with ADHD exhibit deficits in attention domains, including PSI, WMI, and social cognition (Onandia-Hinchado et al., 2021), which is consistent with observations in FASD. Understanding these differences is crucial for accurate identification, intervention, and support for individuals affected by FASD.

ML has been effectively applied in the medical field to diagnose neurological disorders, including ASD (Vakadkar et al., 2021; Bahathiq et al., 2022; Briguglio et al., 2023) and ADHD (Slobodin et al., 2020; Mikolas et al., 2022; Briguglio et al., 2023; Kim et al., 2023). These studies have demonstrated the potential of ML to increase diagnostic accuracy, reduce time to diagnosis and improve reproducibility. For ASD, ML models have been used to identify key traits using sociodemographic, behavioral characteristics, or magnetic resonance imaging (MRI) results, thereby improving and automating the diagnostic process (Vakadkar et al., 2021; Bahathiq et al., 2022; Briguglio et al., 2023). Similarly, ML classifiers for ADHD have been developed based on clinical and psychological data (i.e. attention, impulsiveness, sleep, and emotional disorders) (Slobodin et al., 2020; Mikolas et al., 2022; Kim et al., 2023).

The implementation of ML in FASD diagnosis is crucial due to the complexity and heterogeneity of the disorder. Previous ML studies predicted FAS risk in pregnant drinkers using questionnaires (Oh et al., 2023b) assessing drinking timing, race, ethnicity, alcoholic beverage, prenatal care and pregnancy complications. However, inherent limitations arise due to potential maternal misrepresentation and impracticality when assessing biological mothers' post-adoption. Traditional diagnostic methods for FASD are often challenging, due to multiple factors, like unknown maternal alcohol confirmation, lack of facial dysmorphology or growth impairments, leading to misdiagnosis or delayed diagnosis (Chasnoff et al., 2015). In recent years, research exploring the potential use of ML algorithms for early diagnosing FASD has shown promising results (Suttie et al., 2024). Ehrig et al. used physical characteristics (such as body length and head circumference at birth) and neuropsychological parameters (IQ, behavior, memory) as predictable variables, achieving good levels of accuracy (0.85), precision (0.87), sensitivity (0.91) and AUC (0.93) (Ehrig et al., 2023). Goh et al. (2016) trained their model using CBCL scales, IQ and physical examination, obtaining a sensitivity of 64%−81% and specificity of 78%−80%. Zhang et al. (2019) developed a comprehensive ML framework using eye movements, psychometric tests and brain imaging to predict FASD. Rodriguez et al. (2021) used magnetic resonance imaging to detect PAE. Duarte et al. (2021) trained with NEPSY-II test, saccade eye movement, and diffusion tensor imaging. Furthermore, Lussier et al. (2018) used methylation signatures for FASD classification. Fu et al. (2022) devised a transfer learning approach leveraging extensive facial recognition datasets. Using similar inputs, Blanck-Lubarsch et al. (2022) formulated an automated classification algorithm with 3D facial scans. Our ML model has achieved better accuracy (0.92), precision (0.96), sensitivity (0.92), F1 score (0.94), specificity (0.92), and AUC (0.92) than previous ML algorithms for FASD diagnosis, helping to avoid misdiagnosis in the clinical setting.

This ML prediction aims to be specifically for FASD, thereby distinguishing it from other developmental disorders. Unique FASD indicators like PAE confirmation, distinctive facial features and microcephaly, together with psychometric data, enhance FASD detection. Models have been developed in other pathologies, incorporating clinical and neuropsychological variables, such as ADHD and ASD (Lange et al., 2019; Ehrig et al., 2023). These models have successfully identified these specific pathologies based on a combination of specific variables. Studies from Lange et al. (2019) and Ehrig et al. (2023) used specific parameters that predict FASD compared to ADHD or ASD. These parameters include gestational age, length, weight and head circumference affectation at birth, together with low IQ, socially intrusive behavior, rule-breaking behavior and attention problems. These studies further validate the accuracy of ML in predicting FASD, thereby mitigating the risk of misdiagnosis other neuropathologies.

Based on socio-demographic, clinical, and psychological data from children with FASD the present study has elaborated a common diagnostic model for FASD, obtaining RF algorithm as the best model predictor. We identified important variables for efficient FASD screening, including classic clinical characteristics for diagnosis like maternal alcohol consumption, lip-philtrum, microcephaly, and height and weight impairment. Other significant variables include the WMI, aggressive behavior, IQ, somatic complaints, and depressive problems. WMI, IQ and aggressive behavior are often observed in FASD patients and are considered a significant factor for the diagnostic process (Maya-Enero et al., 2021). However, our study establishes the domains related to somatic complaints and depressive problems, often reported in FASD patients (Mattson et al., 2011), as key diagnostic indicators.

ML models have also been performed for each FASD subtype, identifying specific patterns and enhancing the important variables for precise prediction. Our finding provides a detailed analysis for each specific type of FASD, offering clinicians more precise information for diagnosis and treatment planning.

The ML algorithm that best predicted FAS was XGB and the most important features were traditional physical traits, such as height and weight affectations. Additionally, neuropsychological variables, including FRI, internalizing problems and total problems, play a crucial role in the prediction of FAS, and have previously mentioned its association with FASD (Fagerlund et al., 2011; Popova et al., 2019; Maschke et al., 2021). Furthermore, studies with autism and ADHD found that internalizing problems are also increased, leading to long-term anxiety behavior in adulthood (So et al., 2021; Andersen et al., 2023). These findings suggest that patients primarily affected in these domains may be more likely to exhibit FAS. Interestingly, maternal alcohol consumption, while a significant predictor for pFAS and ARND, does not appear to be a determinant for FAS prediction.

On the other hand, RF model was the best at predicting pFAS, with classical FASD characteristics such as lip-philtrum affectation, confirmed maternal alcohol consumption, IQ, and microcephaly being the most important variables. However, our study showed that neuropsychological variables like PSI, VCI scores, attention problems and thought problems had also impact on pFAS prediction. Therefore, these results suggest that these neuropsychological variables, previously used to diagnose ADHD (Mikolas et al., 2022), together with classical FASD characteristics, may be relevant in predicting pFAS.

Lastly, ARND prediction was best performed by RF algorithm, with maternal alcohol consumption being the most predictable variable. Nevertheless, total problems and attention problems also had some impact on ARND prediction. Previous ML studies also determined that these neurological domains, along with other impairments in CBCL are key factors for bipolar disorder prediction (Uchida et al., 2022).

Conducting a separate machine learning analysis for each type of FASD, once confirmed prenatal alcohol exposure, is potentially beneficial for clinical practice. It allows for a better understanding of FASD subtypes and can contribute to more accurate diagnosis and targeted treatment strategies.

## 5 Conclusions

Our study has carried out significant progress in applying ML to the diagnosis of FASD. ML algorithms effectively diagnose FASD and its subtypes: FAS, pFAS, and ARND. Key variables for efficient FASD screening include classical clinical characteristics (maternal alcohol consumption, lip-philtrum, microcephaly, height and weight impairment) and neuropsychological variables (WMI, aggressive behavior, IQ, somatic complaints, and depressive problems). The best ML algorithm for predicting FAS was XGB, with height, weight affectations, and neuropsychological variables like IQ, internalizing problems, and total problems being the most important features. For pFAS, RF model was the best predictor, considering lip-philtrum affectation, confirmed maternal alcohol consumption, IQ, microcephaly, PSI, VCI, attention problems, and thought problems being the most significant variables. For ARND, the RF algorithm was the best performer, with maternal alcohol consumption, total problems, and attention problems being the most predictable variables. ML improves diagnostic accuracy and enhances understanding of FASD subtypes, leading to early intervention strategies, targeted therapeutic approaches, and ultimately mitigating the secondary disabilities of FASD. This could help the social and health systems for affected individuals and their families, supporting the consensus of the diagnostic criteria. All of this emphasizes the need for public policies to invest in ML integration into diagnostic strategies in order to improve clinical outcomes for FASD individuals. ML models will contribute to the development of more informed public health policies focused on this vulnerable population.

## 6 Limitations

The absence of an ARBD subgroup in our dataset restricts the comprehensiveness of our findings about this FASD subtype. Moreover, self-reported data could introduce bias in variables associated to personal perceptions. External validation on independent datasets is also needed to ensure the robustness of our ML models. Other limitation is related to the confirmation of maternal alcohol consumption, due to incomplete medical records in some adoptees. Additionally, the stress from diagnostic assessments could potentially affect children's performance in neuropsychological tests. Future work could enhance our model by integrating additional data such as magnetic resonance imaging (Rodriguez et al., 2021), NEPSY-II neuropsychological test (Duarte et al., 2021) and eye movement (Zhang et al., 2019) for a more refined FASD diagnosis.

Despite these limitations, our study advances the application of ML in FASD diagnosis, providing a foundation for future research and contributing to the development of more accurate diagnostic tools.

## Data availability statement

The raw data supporting the conclusions of this article will be made available by the authors, without undue reservation.

## Ethics statement

The studies involving humans were approved by Comité Ético de Investigación Clínica Parc de Salut MAR (No. HCB/2021/0459). The studies were conducted in accordance with the local legislation and institutional requirements. Written informed consent for participation in this study was provided by the participants' legal guardians/next of kin.

## Author contributions

AR-T: Formal analysis, Investigation, Methodology, Resources, Writing – original draft, Writing – review & editing, Data curation, Software. EN-T: Data curation, Formal analysis, Investigation, Methodology, Writing – review & editing, Supervision. MV: Conceptualization, Investigation, Resources, Writing – review & editing, Data curation, Formal analysis, Methodology, Visualization. AM: Data curation, Investigation, Methodology, Resources, Visualization, Writing – review & editing. MA: Resources, Data curation, Formal analysis, Methodology, Validation, Writing – review & editing. LA: Data curation, Writing – review & editing, Resources. LM: Supervision, Visualization, Validation, Writing – review & editing, Conceptualization, Funding acquisition, Project administration. ÓG-A: Conceptualization, Funding acquisition, Project administration, Supervision, Validation, Writing – review & editing, Investigation, Resources. VA-F: Project administration, Supervision, Writing – original draft, Investigation, Methodology, Resources, Visualization, Writing – review & editing, Conceptualization, Formal Analysis, Funding acquisition.
