# Supplementary material for: Machine learning algorithms to the early diagnosis of fetal alcohol spectrum disorders
Source: Front Neurosci. 2024 May 6;18:1400933. doi: 10.3389/fnins.2024.1400933 (PMC11131948; doi:10.3389/fnins.2024.1400933)
Supplement: Supplementary file 1 [file Data_Sheet_1.docx]

**SUPPLEMENTARY TABLES**

**Supplementary table 1.** Clinical and physical features of no FASD children versus FASD children (FAS, pFAS and ARND) using chi-square test. Significant differences are bold and were considered when p-value <0,05.

|  | **Premature**  **pvalue** | **Growth disorders**  **pvalue** | **Birth Diseases**  **pvalue** | **Maternal alcohol**  **pvalue** | **Height affectation**  **pvalue** | **Weight affectation**  **pvalue** | **Microcephaly**  **pvalue** | **Short palpebral fissures pvalue** | **Lip-philtrum affectation**  **pvalue** | **Face morphology**  **pvalue** | **Eyes affectation**  **pvalue** | **Upper limbs affectation**  **pvalue** | **Lower limbs affectation**  **pvalue** |
| --- | --- | --- | --- | --- | --- | --- | --- | --- | --- | --- | --- | --- | --- |
| **FAS-NOFAS** | **0,018** | **<0,0001** | 0,062 | **<0,0001** | **<0,0001** | **<0,0001** | **<0,0001** | **0,002** | **<0,0001** | 0,055 | **0,037** | **0,001** | 0,564 |
| **pFAS-NOFAS** | 0,215 | 0,119 | 0,117 | **<0,0001** | **0,022** | **0,002** | **<0,0001** | **0,015** | **<0,0001** | **0,009** | 0,486 | 0,079 | 0,342 |
| **ARND-NOFAS** | 0,485 | **0,040** | **0,052** | **<0,0001** | **0,002** | 0,072 | 0,549 | 0,706 | 0,081 | 0,531 | 0,392 | 0,717 | **0,026** |
| **FAS-pFAS** | 0,182 | **0,014** | 0,567 | 0,739 | **<0,0001** | **<0,0001** | **0,010** | 0,331 | 0,952 | 0,697 | 0,126 | **0,048** | 0,523 |
| **FAS-ARND** | **0,009** | 0,126 | 0,962 | **0,042** | **<0,0001** | **<0,0001** | **<0,0001** | **0,003** | **<0,0001** | 0,262 | **0,011** | **0,011** | 0,134 |
| **pFAS-ARND** | 0,095 | 0,507 | 0,570 | **0,023** | 0,275 | 0,295 | **0,011** | **0,018** | **<0,0001** | 0,100 | 0,154 | 0,247 | 0,096 |

Alcohol-Related Neurodevelopmental Disorder (ARND); Foetal Alcohol Spectrum Disorders (FASD); Foetal Alcohol Syndrome (FAS); partial Foetal Alcohol Syndrome (pFAS).

**Supplementary table 2.** Psychological data using WISC V test of no FASD children versus FASD children (FAS, pFAS and ARND) using Kruskall-Wallis test with Dunn’s correction multiple comparisons test. Significant differences are bold and were considered when p-value <0,05.

|  | **VCI p-value** | **VSI p-value** | **FRI p-value** | **WMI p-value** | **PSI p-value** | **IQ p-value** |
| --- | --- | --- | --- | --- | --- | --- |
| **FAS-NOFAS** | **<0,0001** | **0,011** | **0,002** | **<0,0001** | **0,006** | **<0,0001** |
| **pFAS-NOFAS** | **0,019** | **0,004** | **0,001** | **<0,0001** | **0,003** | **<0,0001** |
| **ARND-NOFAS** | 0,596 | 0,916 | 0,327 | 0,547 | 0,942 | 0,284 |
| **FAS-pFAS** | **0,044** | 0,787 | 0,672 | 0,354 | 0,672 | 0,154 |
| **FAS-ARND** | **0,005** | **0,034** | **0,009** | **0,003** | 0,023 | **0,002** |
| **pFAS-ARND** | 0,194 | **0,026** | **0,005** | **0,009** | 0,023 | **0,030** |

Alcohol-Related Neurodevelopmental Disorder (ARND); Foetal Alcohol Spectrum Disorders (FASD); Foetal Alcohol Syndrome (FAS); partial Foetal Alcohol Syndrome (pFAS).

**Supplementary table 3.** Behavioral data of no FASD children versus FASD children (FAS, pFAS and ARND) using Kruskall-Wallis test with Dunn’s correction multiple comparisons test. Significant differences are bold and were considered when p-value <0,05.

|  | **Thought Problems CBCL**  **p-value** | **Attention Problems CBCL**  **p-value** | **Rule Breaking Behaviour CBCL**  **p-value** | **Aggressive behaviour CBCL**  **p-value** | **Externalising Problems CBCL**  **p-value** | **Total Problems CBCL**  **p-value** | **Anxiety problems CBCL**  **p-value** | **Oppositional defiant problems CBCL**  **p-value** | **Conduct Problems CBCL**  **p-value** | **Obsessive Compulsive problems CBCL**  **p-value** | **Stress problems CBCL**  **p-value** | **Attention problems ASR**  **p-value** |
| --- | --- | --- | --- | --- | --- | --- | --- | --- | --- | --- | --- | --- |
| **FAS-NOFAS** | 0,434 | **0,021** | 0,213 | 0,12 | 0,105 | 0,937 | 0,46 | 0,292 | 0,219 | 0,792 | 0,710 | 0,639 |
| **pFAS-NOFAS** | 0,442 | 0,148 | 0,315 | 0,085 | 0,191 | o,865 | 0,845 | 0,093 | 0,467 | 0,216 | 0,343 | 0,181 |
| **ARND-NOFAS** | **0,035** | **0,02** | **0,018** | 0,142 | **0,045** | **0,008** | **0,009** | 0,078 | 0,066 | **0,049** | **0,001** | 0,381 |
| **FAS-pFAS** | 0,171 | 0,246 | 0,648 | 0,834 | 0,58 | 0,955 | 0,378 | 0,798 | 0,511 | 0,217 | 0,265 | 0,494 |
| **pFAS-ARND** | **0,007** | 0,251 | **0,002** | 0,085 | **0,003** | **0,006** | **0,006** | **0,002** | **0,016** | **0,003** | **<0,0001** | **0,003** |
| **FAS-ARND** | 0,28 | 0,964 | **0,002** | **0,01** | **0,002** | **0,021** | 0,121 | **0,018** | **0,009** | 0,152 | **0,015** | 0,173 |

Alcohol-Related Neurodevelopmental Disorder (ARND); Foetal Alcohol Spectrum Disorders (FASD); Foetal Alcohol Syndrome (FAS); partial Foetal Alcohol Syndrome (pFAS).
